# Causal relationship between smoking and spinal stenosis: Two-sample Mendelian randomization

**DOI:** 10.1097/MD.0000000000039783

**Published:** 2024-09-20

**Authors:** Guang-Hua Deng

**Affiliations:** a Ya’an Hospital of Traditional Chinese Medicine.

**Keywords:** Mendelian randomization, smoking, spinal stenosis

## Abstract

**Objective:**

Currently, the number of patients with spinal stenosis is increasing, and most of the patients are found to have a history of smoking in the clinic. In this study, we used the Mendelian randomization (MR) method to investigate the causal relationship between smoking and spinal stenosis.

**Methods:**

Genetic loci independently associated with smoking and spinal stenosis in people of European ancestry were selected as instrumental variables using pooled data from large-scale genome-wide association studies (GWAS). Three MR analyses, MR-Egger, Weighted median and inverse variance weighting (IVW), were used to investigate the causal relationship between smoking and spinal stenosis. The results were tested for robustness by heterogeneity and multiplicity tests, and sensitivity analyses were performed using the “leave-one-out” method.

**Results:**

The IVW results showed an OR (95% CI) of 2.40 (0.31–18.71), *P* = .403, indicating that there was no causal relationship between smoking and spinal stenosis. And no heterogeneity and multiplicity were found by the test and sensitivity analysis also showed robust results.

**Conclusion:**

In this study, genetic data were analyzed and explored using 2-sample MR analysis, and the results showed that there is a causal relationship between smoking and the occurrence of spinal stenosis, and more studies need to be included.

## 1. Introduction

Spinal stenosis, as a common disease affecting the neurological health of the elderly population, especially more frequent in people over 50 years of age, is characterized by an abnormal narrowing of the space of the spinal canal, which constitutes a compression of the nerve roots or the spinal cord and affects the quality of life of the patients.^[1,2]^ Pathological changes such as degenerative diseases, bone spur formation, and ligament hypertrophy are important causes of the narrowing of the spinal canal space, which leads to compression of the nerves.^[3,4]^ The clinical manifestations are diverse, covering low back pain, radiating pain in the lower limbs, sensory disturbances, and decreased muscle strength, and in severe cases, even affecting the excretory function.^[5–7]^ As a widespread bad habit, the association of smoking with a variety of diseases has been confirmed by a large number of studies.^[8,9]^ Although some studies have suggested that smoking may increase the risk of spinal stenosis,^[10]^ the exact causal link between smoking and spinal stenosis is not yet well documented and needs to be further explored. Given the high prevalence of spinal stenosis and the prevalence of smoking behavior, in-depth exploration of the association between the 2 is important for the prevention and control of spinal stenosis.

Traditional observational studies exploring the association between smoking and spinal stenosis are susceptible to confounding factors and reverse causality, limiting the accuracy of causal inference.^[11]^ Mendelian randomization (MR), a genetic epidemiological strategy, provides a new perspective for assessing potential causal associations between smoking and spinal stenosis by utilizing genetic variations as instrumental variables, such as single nucleotide polymorphisms (SNPs).^[12]^ According to the Mendelian principle of inheritance, genetic variants are randomly assigned at the time of fertilization and are less susceptible to environmental factors, which provides a natural “randomized” design for research and effectively avoids confounding bias.^[13]^ In addition, genotypes remain stable throughout an individual’s life course and are not affected by disease progression, which further eliminates the possibility of reverse causation and enhances the credibility of causal associations.^[14]^

To this end, we conducted a 2-sample MR study to examine the causal association of spinal stenosis in a smoking population. We aimed to provide significant evidence on whether smoking contributes causally to spinal stenosis.

## 2. Data and methods

### 2.1. Data sources

Genome-wide association studies (GWAS) data on smoking and spinal stenosis were obtained via the IEU OpenGWAS project (mr cieu.ac.uk) website. The website was accessed on 2023-09-20.The population source for all final data was Europe, both sexes. Ever smoking (ieu-b-4858) contained 7933,821 SNPs with a sample size of 99,996; spinal stenosis (finn-b-M13_SPINSTENOSIS) contained 16,380,277 SNPs, with 9169 in the experimental group and 164,682 in the control group. This study was a reanalysis of previously collected and published public data and therefore did not require additional ethical approval.

### 2.2. Conditioning of SNP as an instrumental variable

Firstly, the instrumental variables were highly correlated with exposure, with F > 10 as a strong correlation criterion.^[15]^ Second, the instrumental variable is not directly related to the outcome, but only affects the outcome through exposure, i.e. there is no genetic pleiotropy. In this study, the nonexistence of genetic pleiotropy was indicated by a nonzero intercept term (*P* < .05) in the MR-Egger regression model.^[16]^ Finally, instrumental variables were not associated with untested confounding.^[17]^ The human genotype-phenotype association database Phenoscanner V2 was searched for phenotypes associated with the instrumental variables at the genome-wide significance level to determine whether these SNPs were associated with potential risk factors.^[18]^

### 2.3. SNP screening

Significant SNPs were screened from the pooled GWAS data of smoking (*P* < 5 × 10^−8^ was used as the screening condition)^[19]^; the linkage disequilibrium coefficient r2 was set to be 0.001, and the width of linkage disequilibrium region was set to be 10,000 kb to ensure that individual SNPs were independent of each other.^[20]^ The smoking-related SNPs screened above were extracted from the GWAS pooled data of spinal stenosis, while SNPs directly related to outcome indicators were excluded (*P* < 5 × 10^−8^). The *F*-value of each SNP was calculated, and SNPs with weak instrumental variables (*F*-value <10) were excluded.^[21]^ And the human genotype-phenotype association database was queried to screen for potentially relevant risk factor SNPs and exclude them.^[22]^

### 2.4. Causality validation methods

The causal relationship between exposure (smoking) and outcome (spinal stenosis) was mainly verified using inverse variance weighted (IVW) as, supplemented by 3 MR analysis methods, MR-Egger and weighted median, with SNPs as instrumental variables.

### 2.5. Sensitivity analysis

Sensitivity analyses were performed using several methods. First, the Cochran Q test was used to assess the heterogeneity between the individual SNP estimates, and a statistically significant Cochran Q test proved that the analyses were significantly heterogeneous. Second, MR pleiotropy residual sum and outlier (MR-PRESSO) was used to validate the results in the IVW model, to correct for the effect of outliers, and to exclude outliers if they existed and rerun the analysis. Third, the horizontal multiplicity of SNPs was tested using the MR-Egger intercept test (MR-Egger intercept test), and if the intercept term in the MR-Egger intercept test analysis was statistically significant, it indicated that the MR analyses had significant horizontal multiplicity. Fourth, “leave-one-out” sensitivity analyses were performed by removing a single SNP at a time to assess whether the variant drove the association between the exposure and outcome variables. Fifth, funnel plots and forest plots were constructed to visualize the results of the sensitivity analyses. *P* < .05 suggests that there is a potential causal relationship in the MR analyses, which is statistically significant. All statistical analyses were performed using the “TwoSampleMR” package in R software version 4.3.0.

## 3. Results

### 3.1. Instrumental variables

In the present study, 6 SNPs that were strongly associated with ever smoking (*P* < 5 × 10^−8^) without chain disequilibrium (*r*^2^ < 0.001, kb = 10,000) were screened out. Six SNPs that were strongly associated with ever smoking remained after intersecting with the SNPs in the pooled data of the GWAS for spinal stenosis, and excluding those that were directly associated with the outcome indicators. SNP had an *F* value >10, indicating no weak instrumental variables (Table 1). We searched the Human Genotype-Phenotype Association Database and found no potentially relevant risk factor SNPs.

**Table 1:** Information on the final screening of smoking SNPs from GWAS data (n = 6).

ID	SNP	Effect_allele	Other_allele	β	SE	*P*	*F*
1	rs12731986	T	C	−0.0121	0.0022	2.17E−08	30
2	rs12874797	T	C	−0.0194	0.0034	1.06E−08	32
3	rs2011487	A	C	−0.0142	0.0021	3.96E−11	45
4	rs7829715	C	T	−0.0125	0.0021	4.12E−09	35
5	rs846184	T	C	0.0179	0.0032	1.54E−08	31
6	rs9287372	G	A	−0.0148	0.0021	4.05E−12	49

### 3.2. Causal relationship between smoking and spinal stenosis

**Figure 1.**
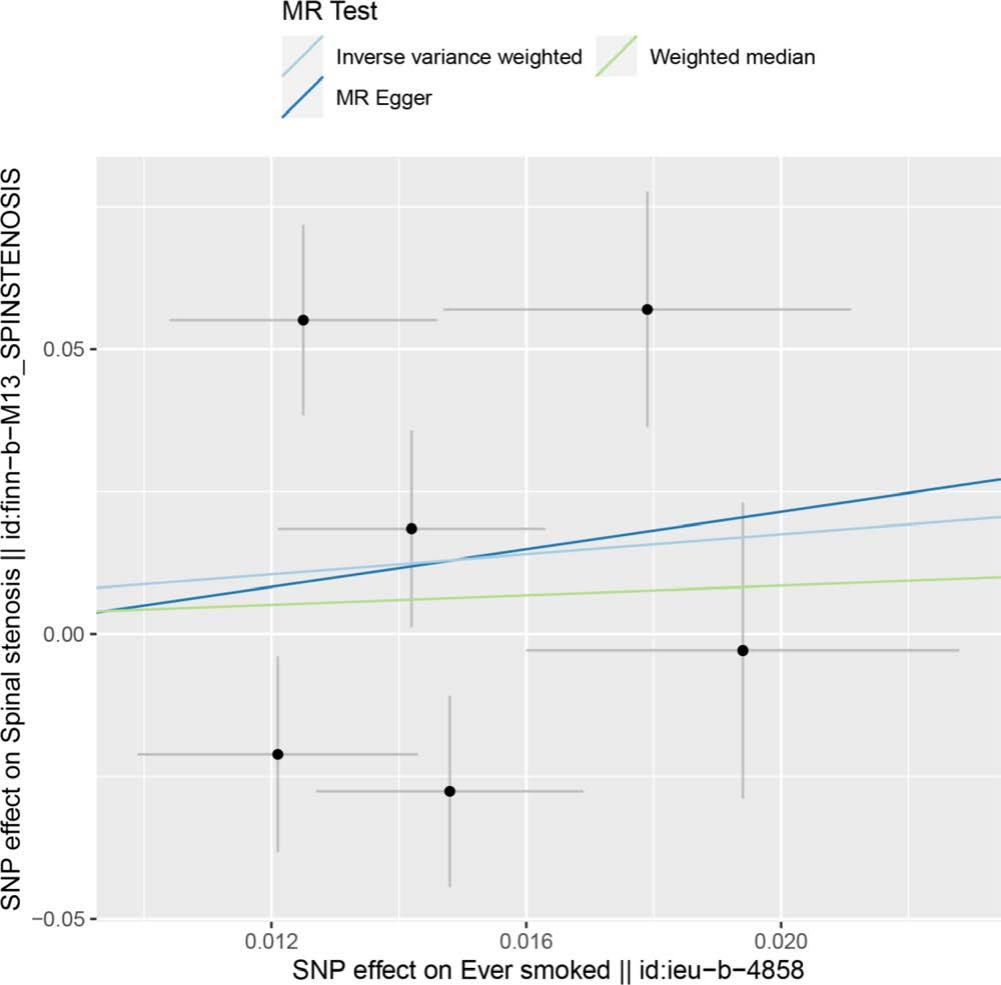
Scatter plot of smoking and spinal stenosis.

**Figure 2.**
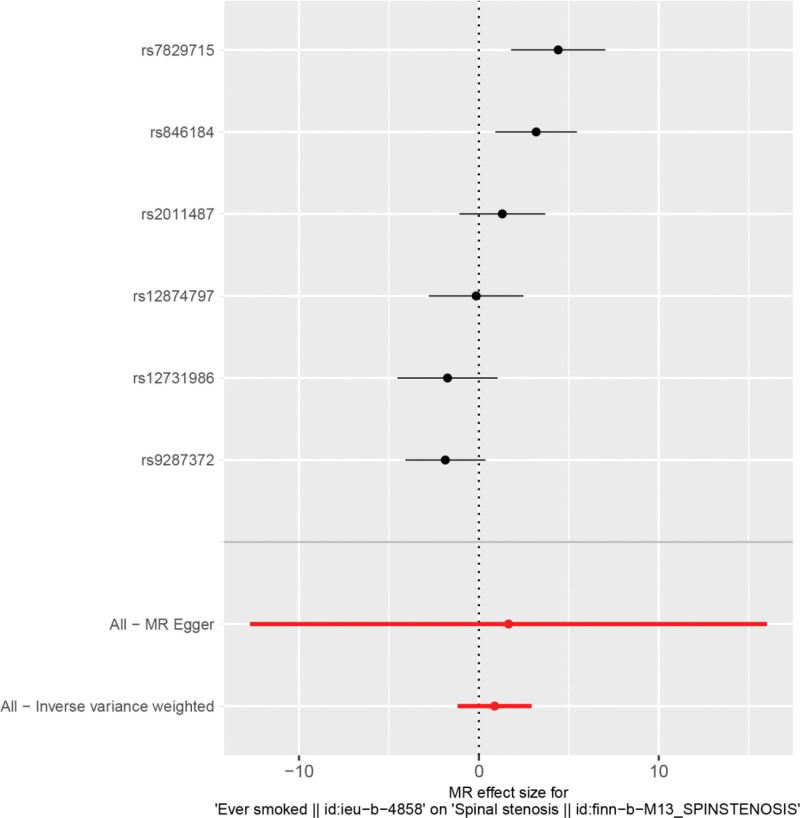
Forest plot of smoking and spinal stenosis.

By MR analysis, the results of inverse variance weighting (IVW), WME, and MR-Egger showed that there was no causal relationship between ever smoking and spinal stenosis. IVW:OR = 2.40, 95% CI = 0.31 to 18.71, *P* = .403; WME:OR = 1.53, 95% CI = 0.35 to 6.72, *P* = .573; MR-Egger:OR = 1.53, 95% CI = 0.573; and MR-Egger:OR = 0.573. Egger:OR = 5.21, 95% CI = 0–9.09e+06, *P* = .833 (Table 2). We can see from both the scatter plot (Fig. 1) and the forest plot (Fig. 2) that ever smoking does not increase the risk of developing spinal stenosis.

**Table 2:** MR regression results of the 3 methods.

Method	β	SE	OR (95% CI)	*P*
IVW	0.875	1.048	2.40 (0.31–18.71)	.403
WME	0.425	0.755	1.53 (0.35–6.72)	.573
MR-Egger	1.650	7.333	5.21 (0–9.09e+06)	.833

### 3.3. Sensitivity analysis

**Figure 3.**
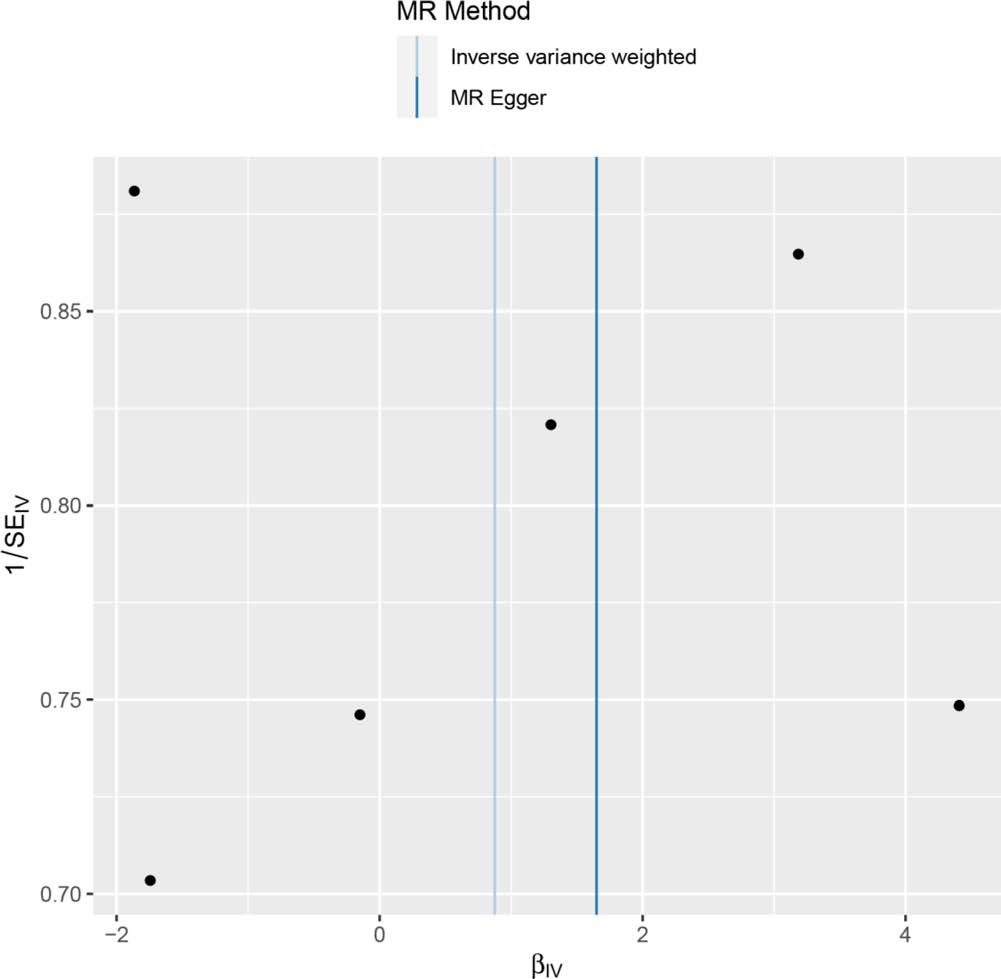
Funnel plot of smoking and spinal stenosis.

**Figure 4.**
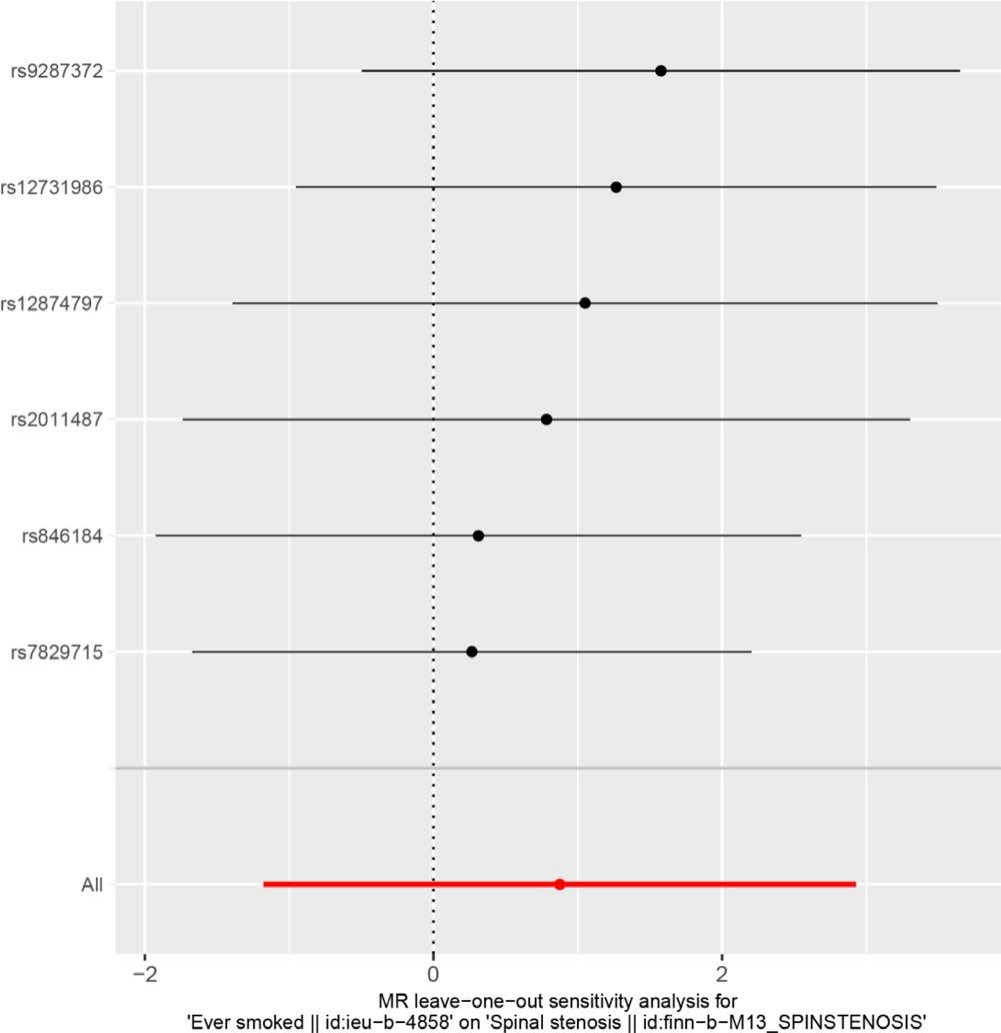
Analysis of smoking and spinal stenosis by the leave-one-out method.

Heterogeneity test, which was performed on the results of ever smoking and spinal stenosis (Cochran *Q* test, *P* = .089), suggested that there was no heterogeneity in the results. A funnel plot was drawn to show the heterogeneous results, as shown in Figure 3. The use of MR-PRESSO was used to screen for SNPs that could lead to heterogeneity, and no SNPs were found to cause heterogeneity in the results. The results of the Global test by MR-PRESSO suggested that there was no pleiotropy between the results of ever smoking and spinal stenosis (*P* = .920). The “leave-one-out” method uses the IVW method by default, and as can be seen in Figure 4, no single SNP would have a large impact on the overall results after excluding any of the SNPs, indicating that the results are robust.

## 4. Discussion

Some studies have suggested that smoking may be an observational risk factor for spinal stenosis, but the causality of this association is unclear. Our MR study aimed to reveal the causal relationship between smoking and spinal stenosis. The 2-sample MR results showed that smoking was not causally associated with the development of spinal stenosis, and the IVW results of a history of previous smoking showed an OR (95 % CI) of 2.40 (0.31–18.71), *P* = .403.

According to relevant studies, it is suggested that the prevalence of spinal stenosis increases with age.^[23]^ Studies have shown that the prevalence of spinal stenosis in people over 60 years of age is about 20% to 30%, while the prevalence is lower in people under 50 years of age.^[24]^ There is a difference in the prevalence of spinal stenosis in men and women. Some studies have shown that the prevalence is slightly higher in male patients than in females, which may be related to the higher frequency of physical labor and exercise in men.^[25]^ The exact cause of spinal stenosis is unknown, but genetic factors are thought to play an important role in its development.^[26]^ The etiology of spinal stenosis is diverse and includes osteophytes, disc herniation, tumors and prolonged repetitive labor.^[10,27–29]^ These factors lead to changes in spinal structure and compression of the spinal cord and nerve roots, which cause spinal stenosis. Individual differences and environmental factors may also have an impact on the etiology. Understanding the etiology of spinal stenosis can help to develop individualized prevention and treatment plans. Smoking may have an impact on the risk of developing spinal stenosis through a number of pathways, such as influencing disc degeneration and osteoporosis. However, these studies are often based on observational study designs, which are retrospective and potentially biased, and do not allow a causal relationship between smoking and spinal stenosis to be established. The present study confirms the noncausal relationship between smoking and spinal stenosis from a genetic perspective. In addition statistical evidence from sensitivity analyses strongly supported our findings.

At the same time there are some limitations of this study. Firstly, as all the data are from a population of European origin, the results do not represent a truly random population sample and are not applicable to other so races. Secondly, although various sensitivity analyses have been performed in this study to test the hypotheses of the MR study, it is difficult to completely rule out horizontal pleiotropy of instrumental variables. Finally, the current sample size of GWAS data is still not large enough, and more in-depth studies using more GWAS data are needed in the future.

## 5. Conclusion

In conclusion, this study used 2-sample MR analysis to analyze and explore the genetic data, and the results showed that the prevalence of spinal stenosis was higher in the smoking population, suggesting that a reduction in the smoking population may reduce the incidence of spinal stenosis.

## Author contributions

**Conceptualization:** Guang-Hua Deng.

**Data curation:** Guang-Hua Deng.

**Formal analysis:** Guang-Hua Deng.

**Funding acquisition:** Guang-Hua Deng.

**Investigation:** Guang-Hua Deng.

**Methodology:** Guang-Hua Deng.

**Project administration:** Guang-Hua Deng.

**Resources:** Guang-Hua Deng.

**Software:** Guang-Hua Deng.

**Supervision:** Guang-Hua Deng.

**Validation:** Guang-Hua Deng.

**Visualization:** Guang-Hua Deng.

**Writing – original draft:** Guang-Hua Deng.

**Writing – review & editing:** Guang-Hua Deng.
